# BMI growth trajectory from birth to 5 years and its sex-specific association with prepregnant BMI and gestational weight gain

**DOI:** 10.3389/fnut.2023.1101158

**Published:** 2023-02-14

**Authors:** Jinting Xie, Yan Han, Lei Peng, Jingjing Zhang, Xiangjun Gong, Yan Du, Xiangmei Ren, Li Zhou, Yuanhong Li, Ping Zeng, Jihong Shao

**Affiliations:** ^1^School of Public Health, Xuzhou Medical University, Xuzhou, Jiangsu, China; ^2^Key Laboratory of Human Genetics and Environmental Medicine, Xuzhou Medical University, Xuzhou, Jiangsu, China; ^3^Key Laboratory of Environment and Health, Xuzhou Medical University, Xuzhou, Jiangsu, China; ^4^Xuzhou Maternal and Child Health Family Planning Service Center, Xuzhou, Jiangsu, China

**Keywords:** BMI-z trajectory, children aged 0–5 years, prepregnant BMI, gestational weight gain, latent class growth model

## Abstract

**Objective:**

The purpose of the study was to identify the latent body mass index (BMI) z-score trajectories of children from birth to 5 years of age and evaluate their sex-specific association with prepregnant BMI and gestational weight gain (GWG).

**Methods:**

This was a retrospective longitudinal cohort study performed in China. In total, three distinct BMI-z trajectories from birth to 5 years of age were determined for both genders using the latent class growth modeling. The logistic regression model was used to assess the associations of maternal prepregnant BMI and GWG with childhood BMI-z growth trajectories.

**Results:**

Excessive GWG increased the risks of children falling into high-BMI-z trajectory relative to adequate GWG (OR = 2.04, 95% CI: 1.29, 3.20) in boys; girls born to mothers with prepregnancy underweight had a higher risk of low-BMI-z trajectory than girls born to mothers with prepregnancy adequate weight (OR = 1.85, 95% CI: 1.22, 2.79).

**Conclusion:**

BMI-z growth trajectories of children from 0 to 5 years of age have population heterogeneity. Prepregnant BMI and GWG are associated with child BMI-z trajectories. It is necessary to monitor weight status before and during pregnancy to promote maternal and child health.

## 1. Introduction

The high prevalence of childhood overweight or obesity is a significant public health issue (1). According to the World Health Organization reports (2), an estimated 5.7% or 38.9 million children under the age of 5 around the world were affected by overweight in 2020. The Report on the Status of Nutrition and Chronic Diseases of Chinese Residents (2020) (3) shows that the prevalence of childhood overweight or obesity was 19% among children aged 6–17 and 10.4% among children under the age of 6. Early childhood overweight or obesity is critical for lifelong health (4, 5). Most prior studies examining the associated gene and environmental determinants of childhood overweight or obesity have focused on childhood BMI at just one point in time (6–8). Compared with the developmental assessments at a single time point, longitudinal child growth trajectories comprehensively evaluate the growth and development level of children from a dynamic perspective and detect abnormal growth in a timely manner (9).

Most studies showed that childhood growth trajectories have population heterogeneity (10–12). The early childhood growth trajectories were proved to be predictive of obesity risk in later life (13), cardiometabolic risk (14, 15), and adult diabetes (16). A recent birth cohort study evaluated childhood BMI z-score trajectories from age of 2 to 18 and showed that preschool age is a critical window that could predict growth patterns during puberty (17). Therefore, it is necessary to closely monitor the early childhood growth trajectories, focusing on those at higher risk of later overweight or obesity status, and helping to target specific groups for early intervention.

Accumulating evidence has supported that prepregnant BMI and gestational weight gain (GWG) may influence childhood overweight or obesity (18–21). Most studies showed that excessive GWG might increase the risk of childhood OWOB (22–24). However, the association between inadequate GWG with childhood BMI status remains unclear (25). Furthermore, there are still gaps in our knowledge regarding the associations of prepregnant BMI and GWG with BMI growth trajectories in early childhood. Therefore, the primary aim of this study was to identify the latent BMI-z growth trajectories of children from birth to 5 years of age in different genders and evaluate their independent association with prepregnant BMI and GWG.

## 2. Methods

### 2.1. Study subjects

The present study was a retrospective longitudinal cohort study. The study was approved by the Ethics Committee of Xuzhou Maternity and Child Health Care Hospital (No.201901).

**Figure 1:**
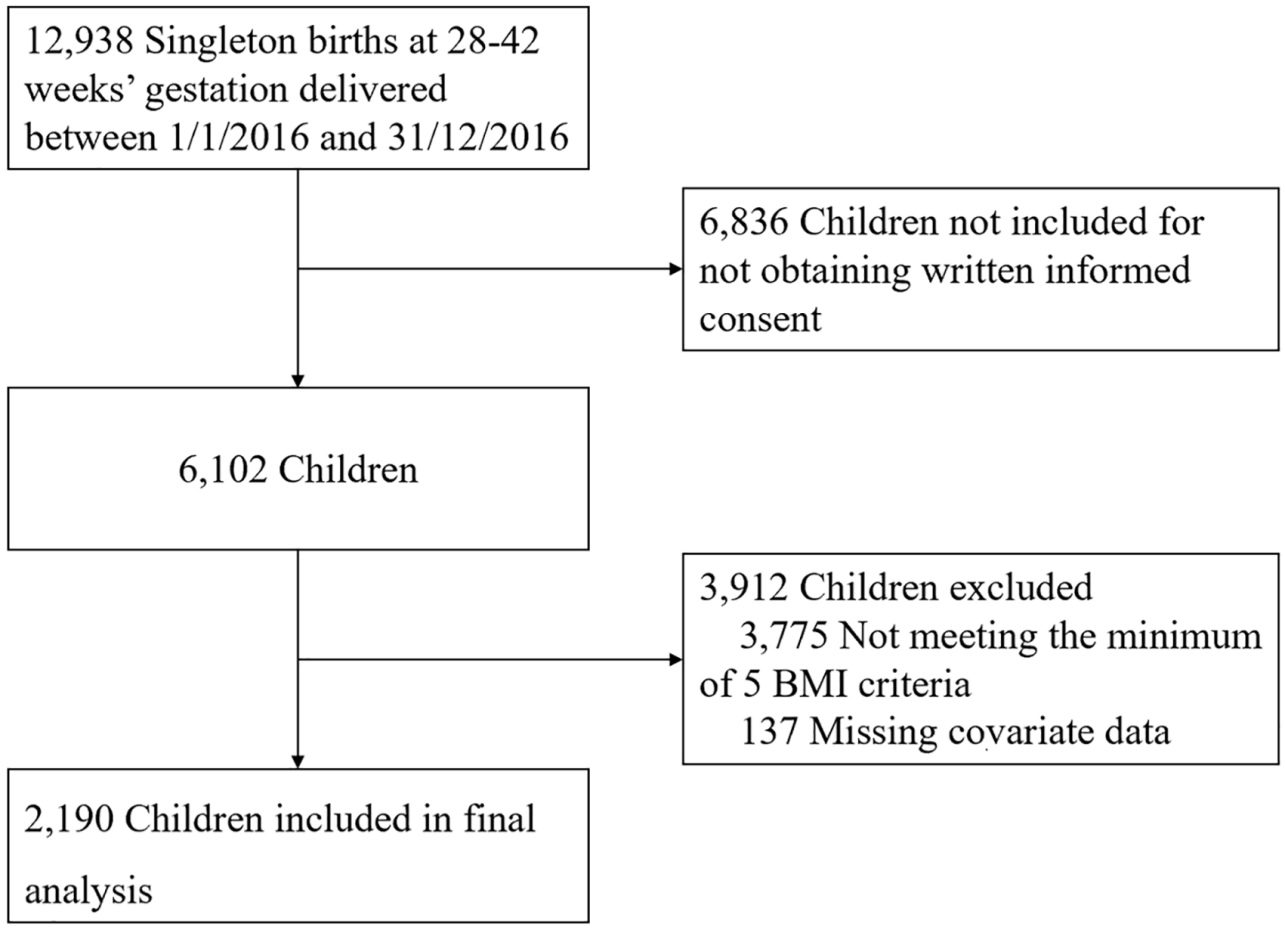
Study cohort derivation.

Participants were singleton offspring born at term in Xuzhou Maternity and Child Health Care Hospital between 1 January 2016 and 31 December 2016. The inclusion criteria included (1) singleton offspring born at 28–42 completed weeks of gestation; and (2) mother–child pairs with recorded information, such as maternal gestational age, education level, prepregnant BMI, GWG, delivery type, child sex, birth weight, feeding mode in 6 months, and children physical check with at least 4 height/length and weight measurement recorded at 1 year (±2 months), 2 years (±2 months), 3 years (±3 months), 4 years (±3 months), and 5 years (±3 months). Exclusion criteria included (1) offspring born with congenital disabilities or postnatal diseases that could interfere with body composition development and (2) offspring with missing covariate data. Figure 1 depicts the study cohort derivation. A total of 2,190 mother–child pairs were enrolled in this study, and written informed consent was obtained from the parents of the subjects at recruitment.

Data of mothers and children were collected retrospectively from the Jiangsu Maternal and Child Health Management Information System, including the maternal and child health information on prenatal, antenatal, and child healthcare electronic records with the standard quality control measures.

### 2.2. Prepregnant BMI and GWG

Height (m) and weight (kg) before pregnancy were collected at enrollment and calculated prepregnant BMI (kg/m^2^). BMI was calculated as weight (kg) divided by square of the length/height (m^2^). According to the BMI standards for Chinese adults (26), mothers were categorized as underweight with a BMI of <18.5 kg/m^2^, adequate weight with 18.5 kg/m^2^ ≤ BMI < 24.0 kg/m^2^, overweight with 24.0 kg/m^2^ ≤ BMI < 28.0 kg/m^2^, and obese with BMI ≥ 28.0 kg/m^2^. Based on their prepregnant BMI, GWG (kg) was calculated by subtracting prepregnancy weight (kg) from maternal weight at delivery (kg) and was categorized based on the standard of recommendation for weight gain during the pregnancy period (WS/T801-2022): underweight mothers (BMI < 18.5 kg/m^2^) who gained 11.0–16.0 kg, normal-weight mothers (18.5 kg/m^2^ ≤ BMI < 24.0 kg/m^2^) gained 8.0–14.0 kg, overweight mothers (24.0 kg/m^2^ ≤ BMI < 28.0 kg/m^2^) gained 7.0–11.0 kg, and obese mothers (BMI ≥ 28.0 kg/m^2^) gained 5.0–9.0 kg were categorized as adequate GWG; mothers who gained weight above or below this criterion were categorized as excessive or inadequate GWG, respectively. The BMI-z values that were more than ±5 were set to missing.

### 2.3. Child BMI-z

Children’s weight and length/height were measured at each annual healthcare visit by trained staff. Body weight was measured using a digital scale (measuring range: 5–150 kg, measurement resolution: 0.1 kg, and measurement accuracy: ±0.3%). Recumbent length was obtained at the first-year and second-year visits, and standing height was obtained for those 3 years or older, all to the nearest 0.1 cm. BMI z-scores (BMI-z) were generated based on the age and sex-specific BMI reference from the WHO Child Growth Standards (2006).

### 2.4. Confounding factors

The potential confounding factors included maternal and children information. Maternal information included household income (￥), mother’s education, prepregnancy BMI (kg/m^2^), age at pregnancy (years), type of delivery, and parity. In terms of children’s information, gestational age of delivery (years), infant feeding mode from birth to 6 months, and time of complementary foods introduction (months) were considered. Child birth weight was categorized as <2.5 kg, 2.5–4 kg, and ≥ 4 kg. Infant feeding mode from birth to 6 months was classified as exclusive breastfeeding (27), mixed feeding, and formula feeding. The time of complementary food introduction was classified as ≤5 months, 6 months, and ≥ 7 months.

### 2.5. Statistical analysis

The latent class growth modeling (LCGM) approach was used to identify the subgroups shared a similar underlying trajectory based on the children’s BMI z-scores with the Mplus 8.0. Model fit indices include Akaike’s Information Criteria (AIC), Bayesian Information Criteria (BIC) and sample size adjusted BIC (aBIC), entropy, and a value of p for bootstrapped likelihood ratio test (BLRT) and Vuong-Lo–Mendell–Rubin likelihood ratio test (VLRT). The smaller the first three indices, the better the model fitting effect. The significant *value of p* for BLRT and VLRT indicates that a model with *k*−1 class should be rejected in favor of a model with k classes. The value of entropy >0.70 and the number of subjects in each trajectory group ≥5% indicate a good model fit. The maximum likelihood robust estimator was used to account for missing data when fitting the trajectories. Three distinct BMI-z trajectories were determined for both genders using LCGM.

Prepregnant BMI and GWG were compared among latent BMI trajectory groups using the chi-square test for proportions and the ANOVA F test for means. Logistic regression models were used to examine the association of prepregnant BMI and GWG with child BMI-z growth trajectory in different genders with the adjustment for the potential confounding factors, including household income, mother’s education, age at pregnancy, type of delivery, parity, birth weight, and time of complementary food introduction. Covariate selection was based on a compulsory entry procedure and other potential confounders identified in the literature (28). Crude and adjusted odd ratios (ORs), along with 95% confidence intervals (CIs), were calculated. Data were analyzed using Statistic Product Service Solutions 23.0. All statistical tests were two-sided, and a *value of p* of <0.05 was considered statistically significant.

## 3. Results

The study population consisted of 2,190 mother–child pairs, of which 1,165 were boys and 1,025 were girls. Table 1 summarizes the maternal and child characteristics of the participants. A higher rate of exclusive breastfeeding in the first 6 months was found in girls than in boys (53.2 vs. 51.1%), and a higher proportion of girls with the time of complementary food introduction at 6 months was found (56.0 vs. 51.9%).

**Table 1:** Population characteristics of participants (*N* = 2,190).

	All children *n* = 2,190	Boy *n* = 1,165	Girl *n* = 1,025	*p*-value
	*n* (%)	*n* (%)	*n* (%)	
Maternal characteristics				
Household income per month, ￥				0.41
<2,500	110 (5.0)	57 (4.9)	53 (5.2)	
2,500–4,000	140 (6.4)	82 (7.0)	58 (5.7)	
≥4,000	1940 (88.6)	1,026 (88.1)	914 (89.2)	
Mother’s level of education				0.52
Junior high or below	296 (13.5)	158 (13.6)	138 (13.5)	
High school/technical secondary school	425 (19.4)	225 (19.3)	200 (19.5)	
Junior college/vocational college	508 (23.2)	284 (24.4)	224 (21.9)	
College degree or above	961 (43.9)	498 (42.7)	463 (45.2)	
Age at pregnancy, years				0.04*
18~	417 (19.0)	209 (17.9)	208 (20.3)	
25~	1,096 (50.0)	569 (48.8)	527 (51.4)	
≥30	677 (30.9)	387 (33.2)	290 (28.3)	
Type of delivery				0.24
Vaginal	1,019 (46.6)	529 (45.4)	491 (47.9)	
Cesarean	1,170 (53.4)	636 (54.6)	534 (52.1)	
Parity				0.06
0	1,375 (62.8)	710 (60.9)	665 (64.9)	
1^+^	815 (37.2)	455 (39.1)	360 (35.1)	
Child characteristics				
Gestational age of delivery, weeks				0.26
<37	106 (4.8)	62 (5.3)	44 (4.3)	
≥37	2084 (95.2)	1,103 (94.7)	981 (95.7)	
Child birth weight, kg				<0.001*
<2.5	35 (1.6)	16 (1.4)	19 (1.9)	
2.5–4	1857 (84.8)	951 (81.6)	906 (88.4)	
≥4	298 (13.6)	198 (17.0)	100 (9.8)	
Infant feeding mode from birth to 6 months				0.49
Exclusive breastfeeding	1,140 (52.1)	595 (51.1)	545 (53.2)	
Mixed feeding	924 (42.2)	498 (42.7)	426 (41.6)	
Formula feeding	126 (5.8)	72 (6.2)	54 (5.3)	
Time of complementary foods introduction, months				0.05
≤5	516 (23.6)	298 (25.6)	218 (21.3)	
6	1,179 (53.8)	605 (51.9)	574 (56.0)	
≥7	495 (34.6)	262 (22.5)	233 (22.7)	

*p < 0.05.

The number of classes and the shape of the BMI z-scores trajectories pattern were estimated according to the data of 1,165 boys and 1,025 girls. Complete BMI-z data were available for 1,043 (89.5%) boys and 917 (89.5%) girls, and the remaining children had one missing BMI-z data. The proportions of missing BMI-z data at each time point are shown in Supplementary Table 1. We tested from one to four possible trajectory classes. Considering the model fit indices, the three-class model was identified as the optimal model for both boys and girls. Supplementary Table 2 shows the fit statistics for the trajectory classes estimated.

**Figure 2:**
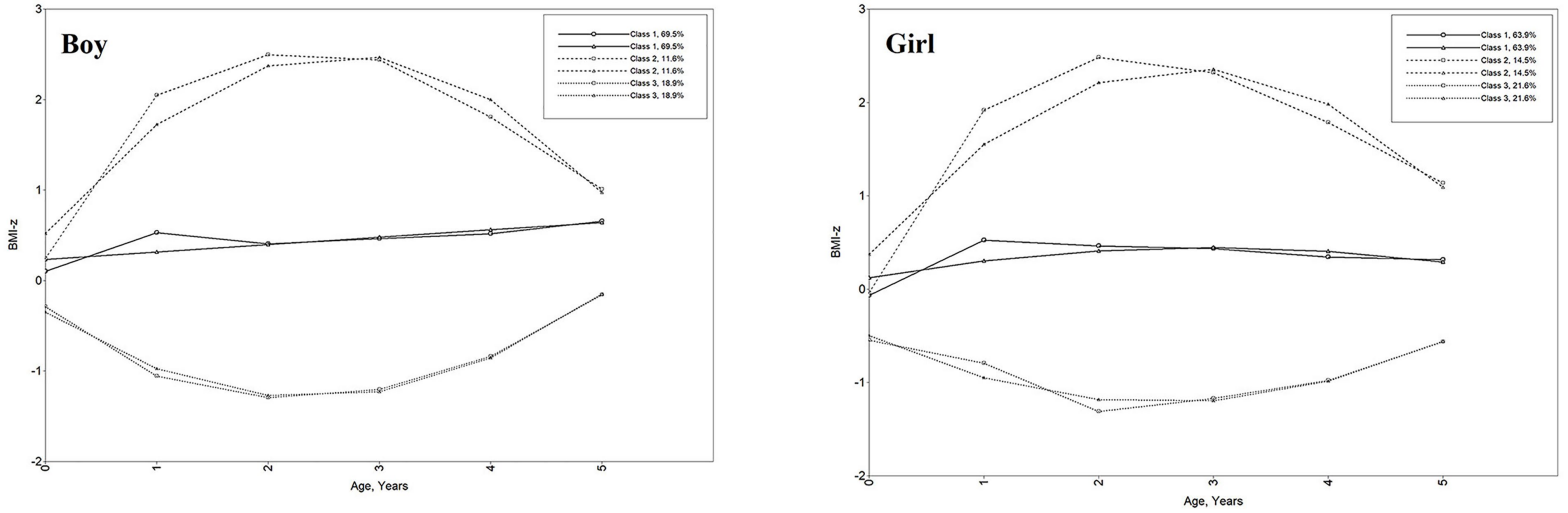
Child BMI-z growth trajectories from birth to 5 years. Class 1: moderate-BMI-z trajectory, Class 2: high-BMI-z trajectory, Class 3: low-BMI-z trajectory. Line of circle symbol represents sample means; line of triangle symbol represents estimated means.

Boys and girls shared similar patterns of growth trajectories but differed in their proportions. For the three latent trajectories, boys were more likely to have stable and moderate growth trajectories than girls. According to the relative position of the estimated three trajectories and combining professional significance, Class 1 was named as “moderate-BMI-z” (69.5% for boys and 63.9% for girls), Class 2 as “high-BMI-z” (11.6% for boys and 14.5% for girls), and Class 3 as “low-BMI-z” (18.9% for boys and 21.6% for girls). Moderate-BMI-z trajectory group represented children who had relatively stable BMI-z scores around 0 with a low increasing trend. The high-BMI-z trajectory group exhibited a relatively high initial BMI-z, a rapid increase until the age of 2 years, and a slight decrease after that, with the overall BMI-z ranging from 1 to 2.5. The low-BMI-z trajectory group was characterized by a relatively low initial BMI-z value, which tends to decrease rapidly until the age of 2 years and then increases slightly into the normal range, with the overall BMI-z ranging from −1.5 to −0.5. Figure 2 shows the BMI-z growth trajectory for trends and sizes of the three kinds of trajectories. Supplementary Table 3 shows the parameter estimation results of the LCGA model for children’s BMI-z growth trajectory.

Table 2 presents the distribution of prepregnant BMI and GWG overall and according to child BMI-z growth trajectory classes stratified for sex. The average prepregnant BMI was 20.83 ± 2.58 kg/m^2^, and the maternal mean GWG was 15.53 ± 5.85 kg. The prevalence of maternal prepregnancy overweight and obesity was 8.4 and 1.6%, respectively, and GWG was classified as excessive for 60.8% of mothers and inadequate for 8.6%. Mothers with excessive GWG were more likely to have children within the high-BMI-z trajectory group among boys (*p* = 0.018). Mothers with prepregnancy underweight were more likely to have children within the low-BMI-z trajectory group among girls (*p* = 0.001).

**Table 2:** Distribution of prepregnant BMI and GWG overall and according to child BMI-z growth trajectory classes stratified for sex.

	Mean ± SD or *n* (%)				
	All	Class 1	Class 2	Class 3	*p*-value
Boys					
Prepregnant BMI, kg/m^2^	20.86 ± 2.57	20.92 ± 2.62	21.06 ± 2.53	20.53 ± 2.38	0.085
Prepregnant BMI category					
Underweight	169 (14.5)	119 (14.7)	17 (12.6)	33 (15.0)	0.564
Adequate weight	876 (75.2)	607 (74.9)	99 (73.3)	170 (77.3)	
Overweight	101 (8.7)	69 (8.5)	17 (12.6)	15 (6.8)	
Obesity	19 (1.6)	15 (1.9)	2 (1.5)	2 (0.9)	
GWG, kg	15.32 ± 5.82	15.25 ± 5.88	16.18 ± 5.92	15.07 ± 5.44	0.172
GWG					
Adequate	357 (30.6)	265 (32.7)	31 (23.0)	61 (27.7)	0.018
Inadequate	105 (9.0)	72 (8.9)	7 (5.2)	26 (11.8)	
Excessive	703 (60.3)	473 (58.4)	97 (71.9)	133 (60.5)	
Girls					
Prepregnant BMI, kg/m^2^	20.80 ± 2.58	20.81 ± 2.55	21.28 ± 2.69	20.44 ± 2.55	0.009
Prepregnant BMI category					
Underweight	156 (15.2)	92 (14.0)	12 (8.1)	52 (23.5)	0.001
adequate weight	768 (74.9)	501 (76.5)	116 (77.9)	151 (68.3)	
Overweight	84 (8.2)	50 (7.6)	19 (12.8)	15 (6.8)	
Obesity	17 (1.7)	12 (1.8)	2 (1.3)	3 (1.4)	
GWG, kg	15.77 ± 5.88	15.70 ± 5.59	15.08 ± 5.36	16.41 ± 6.91	0.095
GWG					
Adequate	312 (30.4)	201 (30.7)	42 (28.2)	69 (31.2)	0.705
Inadequate	84 (8.2)	52 (7.9)	10 (6.7)	22 (10.0)	
Excessive	629 (61.4)	402 (61.4)	97 (65.1)	130 (58.8)	

Table 3 shows the independent association of prepregnant BMI and GWG with child BMI-z growth trajectory. After adjusting for household income, mother’s education, age at pregnancy, type of delivery, parity, birth weight, time of complementary food introduction, and defining the moderate-BMI-z trajectory group as the reference category, boys of maternal excessive GWG were more likely to have high-BMI-z trajectory than their adequate GWG counterparts (OR = 2.04, 95% CI:1.29, 3.20); girls born to the mothers with prepregnancy underweight were more likely to have low-BMI-z trajectory than girls born to the mothers with prepregnancy adequate weight (OR = 1.85, 95% CI: 1.22, 2.79).

**Table 3:** The independent associations of prepregnant BMI and GWG on child BMI-z growth trajectory OR (95%CI).

	Boys		Girls	
	High-BMI-z trajectory	Low-BMI-z trajectory	High-BMI-z trajectory	Low-BMI-z trajectory
	OR (95%CI)	OR (95%CI)	OR (95%CI)	OR (95%CI)
Model 1^a^				
Prepregnant BMI category				
Adequate weight	1.00	1.00	1.00	1
Underweight	1.02 (0.58, 1.80)	0.97 (0.63, 1.50)	0.58 (0.30, 1.11)	**1.88 (1.26, 2.81)**^b^
Overweight	1.52 (0.86, 2.70)	0.78 (0.43, 1.39)	1.64 (0.93, 2.89)	0.99 (0.54, 1.82)
Obesity	0.75 (0.17, 3.34)	0.48 (0.11, 2.11)	0.71 (0.16, 3.22)	0.83 (0.23, 2.98)
Gestational weight gain				
Adequate	1	1	1	1
Inadequate	0.83 (0.35, 1.98)	1.56 (0.92, 2.66)	0.95 (0.44, 2.02)	1.16 (0.65, 2.06)
Excessive	**1.76 (1.14, 2.72)**	0.48 (0.11, 2.11)	1.07 (0.71, 1.61)	1.06 (0.75, 1.51)
Model 2				
Prepregnant BMI category				
Adequate weight	1.00	1.00	1.00	1
Underweight	1.10 (0.60, 2.00)	0.96 (0.61, 1.51)	0.63 (0.33, 1.23)	**1.85 (1.22, 2.79)**
Overweight	1.41 (0.76, 2.59)	0.84 (0.46, 1.54)	1.46 (0.80, 2.65)	0.94 (0.50, 1.76)
Obesity	0.39 (0.08, 1.84)	0.37 (0.08, 1.72)	0.57 (0.12, 2.72)	0.84 (0.23, 3.08)
Gestational weight gain				
Adequate	1.00	1.00	1.00	1
Inadequate	0.84 (0.35, 2.03)	1.54 (0.89, 2.69)	0.72 (0.33, 1.61)	1.28 (0.71, 2.32)
Excessive	**2.04 (1.29, 3.20)**	1.41 (0.99, 2.01)	1.18 (0.77, 1.80)	1.07 (0.75, 1.52)

^a^Model 1, Crude model; Model 2, Adjusted for household income, mother’s education, age at pregnancy, type of delivery, parity, gestational age of delivery, birth weight, time of complementary foods introduction. ^b^The bold data indicates the value of p less than 0.05.

## 4. Discussion

This study was conducted to identify the childhood BMI-z trajectories from birth to 5 years among children born at term in Xuzhou Maternity and Child Health Care Hospital between 1 January 2016 and 31 December 2016 in different genders and to assess the association between BMI-z trajectories with prepregnant BMI and GWG. In total, three main findings are worthy of further attention and discussion. Data from this retrospective longitudinal cohort study showed that childhood BMI-z trajectories from birth to 5 years could be classified into three latent groups for both boys and girls, characterized as moderate-BMI-z trajectory group, high-BMI-z trajectory group, and low-BMI-z trajectory group. Prepregnant BMI and GWG were significantly associated with childhood BMI-z trajectories. Excessive GWG predicted the increased risk for the high-BMI-z trajectory group for boys, and prepregnancy underweight predicted the increased risk for the low-BMI-z trajectory group for girls.

With the application of longitudinal data analysis methods in childhood growth trajectories, accumulating studies have documented the potential heterogeneity of childhood growth trajectories (29, 30). The growth trajectory of early childhood is particularly important. Children at risk for overweight and obesity may have unique developmental trajectories during early childhood (31), which may influence the subsequent development of overweight or obesity and other health issues (14, 32). The classification and description of early childhood BMI-z growth trajectory in previous studies (33–36) can be roughly summarized into three types, including stable-moderate BMI-z growth trajectory, stable-low BMI-z growth trajectory, and stable-high BMI-z growth trajectory, which are consistent with the trajectories observed in our study. Furthermore, similar to these studies, it was concluded that children with stable-moderate BMI-z growth trajectory were in the majority, approximately 60% account, with the average BMI-z score range of around 0. However, Zhang et al. (37) reported four latent BMI-z growth trajectory patterns from birth to the age of 60 months. In addition to the three categories mentioned above, a catch-up BMI-z growth trajectory was also identified. The reason this result differs from our study may be due to the different fitting methods used (38). In future research, it would be beneficial to examine the application of different longitudinal data analysis methods to childhood growth trajectories.

In our analysis, after adjusting for the potential confounders, we found that boys of mothers with excessive GWG were significantly associated with an increased risk of high-BMI-z trajectory from birth to 5 years of age. Similarly, a retrospective longitudinal cohort study of 71,892 children suggested that children’s high and increasing BMI trajectories were modestly associated with excessive GWG (28). However, the index used in this study to fit the growth trajectory of children is the raw BMI value, and the timeframe for the trajectory is 2–6 years old. Compared with the BMI trajectory, the BMI-z trajectory can better reflect the change in BMI values relative to their peers. Additionally, Montazeri et al. (39) found that excessive GWG was positively associated with the BMI trajectory of higher birth size and subsequent accelerated BMI gain, and inadequate GWG was associated with the BMI trajectory of lower birth size and slower BMI gain. However, our study did not observe a significant association between inadequate GWG and the low-BMI-z trajectory after adjustment. This may be due to the differences in the timeframe and classification of childhood growth trajectories. More in-depth studies are required to determine how inadequate GWG affects childhood growth trajectory.

Previous studies have reported positive associations between maternal prepregnant obesity and child high BMI growth trajectory (24, 40), which were consistent with the results of our study, though not statistically significant. Our study found that maternal prepregnant underweight predicted the increased risk for the low-BMI-z trajectory group for girls, which may be explained mainly by long-term changes in fetal endocrine and metabolic disorders (41).

There are several strengths in our study. First, multiple assessment points of BMI-z score were collected to identify the childhood growth trajectories with the LCGM approach, revealing the potential heterogeneity of growth trajectories in early childhood. Second, the study was conducted based on the Jiangsu Maternal and Child Health Management Information System to collect maternal and children’s health data, which was electronically recorded with the standard quality control measures. Third, GWG was classified according to the standard of recommendation for weight gain during the pregnancy period (WS/T801-2022), which was based on the BMI of Chinese adults as the tangent point and more suitable for evaluating maternal weight status of Chinese women than the Institute of Medicine guidelines in 2009. Finally, a relatively large sample size was used in the study. It took comprehensive covariates into inclusion in evaluating the association between prepregnant BMI and GWG with childhood growth trajectories.

Nevertheless, several limitations should be mentioned as well. First, the retrospective cohort study was used in this study, and the credibility of the evidence is insufficient. Hence, more prospective studies are needed to corroborate the results of this study in the future. Second, the information on energy balance-related behavior was not collected and adjusted for in our study, which needs to be considered in the future.

## 5. Conclusion

The BMI-z trajectory from birth to 5 years of age was identified as three latent groups both for boys and girls with the approach of LCGM. Excessive GWG is associated with the increased risk for the high-BMI-z trajectory group for boys, prepregnancy underweight predicted the increased risk for the low-BMI-z trajectory group for girls. Pre-school age is the key window for the formation of trajectory patterns. Maternal weight should be managed precisely, and physical surveillance and intervention should be carried out for children at high risk of obesity, which can help to move the threshold of prevention and control of childhood overweight or obesity forward.

## Data availbility statement

The raw data supporting the conclusions of this article will be made available by the authors, without undue reservation.

## Author contributions

JS, YH, and LP designed and conceptualized the study. XG, YH, and PZ were responsible for the methodology. JX, YH, and JZ conducted the formal analysis. YD, JZ, and JX were the investigators. JX and JZ prepared the original draft of the manuscript. JS, XR, LZ, and YL reviewed and edited the manuscript. All authors contributed to the article and approved the submitted version.

## Funding

This study was supported by the National Natural Science Foundation of China (no. 82204056), the Jiangsu Provincial Maternal and Child Health Research Project in 2018 (F201805), the Key Lab of Human Genetics and Environmental Medicine, School of Public Health, Xuzhou Medical University, and the Key Lab of Environment and Health, School of Public Health, Xuzhou Medical University.

## Conflict of interest

The authors declare that the research was conducted in the absence of any commercial or financial relationships that could be construed as a potential conflict of interest.

## Publisher’s note

All claims expressed in this article are solely those of the authors and do not necessarily represent those of their affiliated organizations, or those of the publisher, the editors and the reviewers. Any product that may be evaluated in this article, or claim that may be made by its manufacturer, is not guaranteed or endorsed by the publisher.
