# Supplementary material for: BMI growth trajectory from birth to 5 years and its sex-specific association with prepregnant BMI and gestational weight gain
Source: Front Nutr. 2023 Feb 14;10:1101158. doi: 10.3389/fnut.2023.1101158 (PMC9971005; doi:10.3389/fnut.2023.1101158)
Supplement: Supplementary file 1 [file Table_1.DOCX]

**Supplemental Table 1** Frequency and proportion of missing BMI-z data at each time point stratified for sex.

| Missing BMI-z data at each timepoint | Boys | |  | Girls | |
| --- | --- | --- | --- | --- | --- |
|  | n | % |  | n | % |
| Birth | 28 | 2.4 |  | 24 | 2.3 |
| Year 1 | 37 | 3.2 |  | 31 | 3.0 |
| Year 2 | 14 | 1.2 |  | 16 | 1.6 |
| Year 3 | 11 | 0.9 |  | 12 | 1.2 |
| Year 4 | 20 | 1.7 |  | 8 | 0.8 |
| Year 5 | 12 | 1.0 |  | 17 | 1.7 |
