# Supplementary material for: BMI growth trajectory from birth to 5 years and its sex-specific association with prepregnant BMI and gestational weight gain
Source: Front Nutr. 2023 Feb 14;10:1101158. doi: 10.3389/fnut.2023.1101158 (PMC9971005; doi:10.3389/fnut.2023.1101158)
Supplement: Supplementary file 2 [file Table_2.docx]

**Supplemental Table 2** Comparison indices for models with different numbers of latent classes.

| Class | Percentage of subjects per class | AIC | BIC | aBIC | Entropy | VLRT | BLRT |
| --- | --- | --- | --- | --- | --- | --- | --- |
| Boys |  |  |  |  |  |  |  |
| 1 | 100.0 | 22846 | 22897 | 22865 |  |  |  |
| 2 | 37.0/63.0 | 21982 | 22053 | 22008 | 0.690 | ＜0.001 | ＜0.001 |
| **3** | **12.5/67.3/20.2** | **21522** | **21613** | **21556** | **0.811** | **＜0.001** | **＜0.001** |
| 4 | 8.3/36.2/47.0/8.5 | 21379 | 21491 | 21421 | 0.744 | 0.012 | ＜0.001 |
| Girls |  |  |  |  |  |  |  |
| 1 | 100.0 | 19636 | 19685 | 19654 |  |  |  |
| 2 | 44.4/55.6 | 18737 | 18806 | 18761 | 0.699 | ＜0.001 | ＜0.001 |
| **3** | **15.3/62.7/22.0** | **18321** | **18410** | **18353** | **0.785** | **0.0317** | **＜0.001** |
| 4 | 10.6/45.9/39.8/3.7 | 18110 | 18218 | 18148 | 0.796 | 0.0018 | ＜0.001 |
