# Supplementary material for: BMI growth trajectory from birth to 5 years and its sex-specific association with prepregnant BMI and gestational weight gain
Source: Front Nutr. 2023 Feb 14;10:1101158. doi: 10.3389/fnut.2023.1101158 (PMC9971005; doi:10.3389/fnut.2023.1101158)
Supplement: Supplementary file 3 [file Table_3.docx]

**Supplemental Table 3** Parameter estimation results of LCGA model for child BMI-z growth trajectory.

|  | Child BMI-z trajectory | Estimated mean | Standard Error | *P*-value |
| --- | --- | --- | --- | --- |
| Boys | Class 1 moderate-BMI-z | （n=810，*p*=69.5%） | | |
|  | *α*_1_ | 0.234 | 0.046 | ＜0.001 |
|  | *β*1 1 | 0.082 | 0.054 | 0.128 |
|  | *β*2 1 | 0.000 | 0.009 | 0.986 |
|  | Class 2 high-BMI-z | （n=135，*p*=11.6%） | | |
|  | *α*_1_ | 0.519 | 0.146 | ＜0.001 |
|  | *β*1 2 | 1.485 | 0.110 | ＜0.001 |
|  | *β*2 2 | -0.279 | 0.025 | ＜0.001 |
|  | Class 3 low-BMI-z | （n=220，*p*=18.9%） | | |
|  | *α*_1_ | -0.350 | 0.101 | 0.001 |
|  | *β*1 3 | -0.793 | 0.097 | ＜0.001 |
|  | *β*2 3 | 0.167 | 0.019 | ＜0.001 |
| Girls | Class 1 moderate-BMI-z | （n=655，*p*=63.9%） | | |
|  | *α*_1_ | 0.123 | 0.068 | 0.071 |
|  | *β*1 1 | 0.218 | 0.090 | 0.015 |
|  | *β*2 1 | -0.037 | 0.016 | 0.023 |
|  | Class 2 high-BMI-z | （n=149，*p*=14.5%） | | |
|  | *α*_1_ | 0.374 | 0.131 | 0.004 |
|  | *β*1 2 | 1.435 | 0.162 | ＜0.001 |
|  | *β*2 2 | -0.258 | 0.027 | ＜0.001 |
|  | Class 3 low-BMI-z | （n=221，*p*=21.6%） | | |
|  | *α*_1_ | -0.499 | 0.158 | 0.002 |
|  | *β*1 3 | -0.561 | 0.129 | ＜0.001 |
|  | *β*2 3 | 0.110 | 0.025 | ＜0.001 |

*α*_k_ represents intercept term；*β*1 k and *β*2 k represent slope factor and coefficient of quadratic term.
